# How will we prepare for an uncertain future? The value of open data and code for unborn generations facing climate change

**DOI:** 10.1098/rspb.2024.1515

**Published:** 2025-02-12

**Authors:** Dylan G. E. Gomes

**Affiliations:** ^1^ U.S. Geological Survey, Forest and Rangeland Ecosystem Science Center, Seattle, WA 98195, USA; ^2^ Former affiliation: National Academy of Sciences NRC Postdoctoral Research Associateship, Northwest Fisheries Science Center, National Marine Fisheries Service, National Oceanic and Atmospheric Administration, Seattle, WA 98112, USA

**Keywords:** open science, data archiving, transparency, reproducible, reproducibility

## Abstract

As the impacts of climate change continue to intensify, humans face new challenges to long-term survival. Humans will likely be battling these problems long after 2100, when many climate projections currently end. A more forward-thinking view on our science and its direction may help better prepare for the future of our species. Researchers may consider datasets the basic units of knowledge, whose preservation is arguably more important than the articles that are written about them. Storing data and code in long-term repositories offers insurance against our uncertain future. To ensure open data are useful, data must be FAIR (Findable, Accessible, Interoperable and Reusable) and be complete with all appropriate metadata. By embracing open science practices, contemporary scientists give the future of humanity the information to make better decisions, save time and other valuable resources, and increase global equity as access to information is made free. This, in turn, could enable and inspire a diversity of solutions, to the benefit of many. Imagine the collective science conducted, the models built, and the questions answered if all of the data researchers have collectively gathered were organized and immediately accessible and usable by everyone. Investing in open science today may ensure a brighter future for unborn generations.

## 1. Introduction

Climate change is undeniably altering Earth, from the highest peaks to the world’s oceans [1]. Humans have been aware of changes to the global climate for over a century [2,3]. The relatively slow response in adjusting emissions [4,5] suggests that we may be tackling the problem of human-induced climate change for an extended period of time, likely long after the year 2100—a date that is commonly the end of future climate change projections [6]. While humans should continue to reduce greenhouse gas emissions, mitigate the effects of those emissions already released, and continue research to understand climate change and climate impact in the immediate term, we also need to begin to think on a broader temporal scale and prepare for our future battles with such persistent challenges.

What climate change questions will future researchers be trying to answer in the year 2100? This question is impossible to answer precisely, but contemporary scientists have some control in how prepared future researchers are for the uncertain effects of climate change. That is, knowledge workers can leave behind masses of organized and detailed information, which can allow the future of humanity to synthesize information in novel ways [7] and make more informed decisions [8–10]. However, there are currently many systemic issues that make synthesis and informed decision-making more difficult, such as improperly analysed data [11,12], non-reproducible research [13], fabricated results [14,15], publication bias [16,17] and a general lack of available data [18]. While open data do not eliminate these systemic issues, they allow for proper reanalysis [19], assessment of reproducibility [20], identification of fabricated results [21,22] and documentation of publication bias [23], along with providing immediately available data for meta-analyses focused on generating new insights [7,24,25].

Why not make our data and/or code the most useful it can be, potentially long into the future? There are, of course, many legitimate hesitations in data sharing [26–29], including the potential unethical or discriminatory misuse of data [30,31]. The historic abuse of people and their information, for example, makes an obvious case for respecting indigenous data sovereignty [32]. Data collected on Indigenous Peoples can be overly focused on highlighting a narrative of deficit (i.e. portraying Indigenous communities through perceived deficiencies or lack of capabilities), and making these data open can cause more harm than benefit as this narrative is perpetuated [33]. CARE Principles (Collective benefit, Authority to control, Responsibility, Ethics) can help guide best practices for governance and stewardship of Indigenous data [34]. There are many other types of sensitive data (e.g. individual health records, personal financial information and interview transcripts) that generate privacy and security concerns and which could be (mis)used out of context or without further ethical approval if they were made openly available [35]. In a more extreme example, termed dual-use research of concern, open genetic information could be used for viral engineering in a biological warfare or terrorism context [36]. In many of these cases, it is important to note that data often should not be made open. Each case should be handled with care and available resources for working with sensitive data be consulted [37–40].

In the majority of cases of data sharing hesitations in the biological sciences, there are other barriers, such as a lack of time, not knowing where to store open data or simply not wanting to share data or code [27,29], that can be more easily overcome. Many of these barriers are being lowered and the benefits of open data and code outweigh the costs to individual researchers [41]. Benefits include increased citation rates, more organized and efficient projects, increased collaboration opportunities, positive reviews and enhanced ability to find errors before, during and after publication [28,29,42–46], but work from Berberi & Roche [47] suggests that open data policies, by themselves, do not necessarily increase article retractions or corrections. Yet, the benefits do not stop at the individual researcher. Society could profit from open science for generations to come [48–52].

## 2. The collective advantages today

Many individuals are already familiar with the power of open data and open science tools, as it leads to more informed decision-making and saves time and other valuable resources [48,49,52–54]. It is not difficult to imagine the countless ways that open datasets have sped up knowledge production [55–57], lowered costs [48] and have advanced innovation for the benefit of society, not just individual research projects [58]. As common examples, researchers often use open datasets providing global air or sea surface temperatures and satellite imagery in scientific work [59,60]. Freely available satellite imagery has been used in countless contexts to aid in our collective understanding of the natural world. With such tools, we have been able to monitor water use and availability, plant and animal habitat availability, sustainable development and many other land use changes over time [61–63].

Yet, simply sharing data is not enough [55]. For data to be useful they must be FAIR (Findable, Accessible, Interoperable and Reusable; [64]). That is, they must be stored in openly available digital repositories in machine-readable (data that are structured in a way that computers can automatically read and process, without human intervention), non-proprietary formats (i.e. accessible to all with a computer, not just those with access to proprietary software). Repositories should include enough metadata for a naive reader to be able to interpret how, where, when and why the data were collected (including units of measurement and relevant caveats). While complying with FAIR guidelines can seem daunting at first, especially for those who are inexperienced in open science practices, there are many resources available [29,64]. For example, there has been considerable effort into developing many metadata templates, standards, protocols and guidelines [65–70] that can be followed until individual workflows become common practice and a part of the normal publishing process.

Long-term repositories that mint digital object identifiers (DOI) for data and code (e.g. Zenodo, Dryad, Open Science Framework) ensure longevity, whereas including data or code as journal supplementary materials or linking articles to GitHub accounts via URLs (i.e. not archiving through the GitHub–Zenodo integration) is more likely to be short-lived as URLs are almost certain to be broken not far into the future [71,72]. Attaching a DOI to data and code has the additional advantage of allowing for the direct citation of those data and code when they are reused for other purposes.

Despite these useful guidelines, there are still practical decisions that individuals must make. Exactly what form of ‘raw’ data will be useful to others will depend on the subdiscipline and the new research questions. For example, active acoustics in fishery research produces many large sound files that require substantial processing before biological information can be extracted [73,74]. Raw sound files are large and cumbersome, such that they are less likely to be shared directly, relative to the processed data (e.g. estimates of biomass; [75]). While the processed data are both easier to share and re-use for many applications, raw sound files are still useful, as they can be re-processed in other ways to assess completely different questions, providing a valuable resource (e.g. building a timeseries for a different target species; [76]). Thus, we should strive to archive all our data [77] within reason, even if we cannot imagine what future scientists will use them for. With this said, larger datasets can present significant archiving and re-use challenges [78–81]. Some repositories have relatively high storage limits that will suit many research project needs, and other solutions exist for larger datasets, such as bundling into smaller datasets such that each is within the storage limit [82,83]. However, some datasets are so large that they impose high financial and environmental costs to be retained and preserved. In these field-specific cases, decisions must be made about what types of data are important enough to warrant the resource-intensive process of retaining and preserving these data.

Not only does open data benefit scientific progress, open code does as well. Like open data, the usefulness of open code depends on how it is shared. That is, it ideally needs to be adequately described, reviewed, quality controlled and operable when shared [84–86]. Many researchers directly benefit from others who build, and maintain, freely available software that we use for data storage and sharing, processing, visualization, analysis and collaboration [54,87–91]. With advances in open software availability and cheap computing power, free modelling tools are becoming increasingly complex, which can increase our ability to build an accurate representation of the world around us [92–94].

Ecosystem models, for example, are powerful simulation-based process models which can help in assessing potential future management and climate scenarios in a holistic ecosystem-based framework. Yet, complex system models are inherently data-hungry, and model predictions are often limited by the available data [95]. There are several open or publicly available resources that can aid in ecosystem model development and parameterization [96–98], yet entire-system model-building efforts are still costly and time-consuming, as many sources of information are missing or difficult to obtain [8,99].

From my own recent experience re-parameterizing an end-to-end ecosystem model [100], obtaining datasets from collaborators within a common institution took an average of nearly 100 days from the first data request and required over 80 e-mails per dataset to obtain the data, clarify metadata and outline dataset caveats (table 1). Two datasets took nearly 1 year to retrieve, despite the fact that the U.S. Government *requires* that these taxpayer-funded datasets are made publicly available [58,102,103]. While communication during a data transfer process can clarify many caveats and misconceptions about a dataset, and thus is not inherently undesirable, substantial delays in integrating such information causes increased costs in providing new knowledge and can unnecessarily postpone overdue conservation management actions and decision-making [104,105].

**Table 1.** Effort required gathering in-house (National Oceanic and Atmospheric Administration [NOAA] Fisheries-produced) data, during my postdoctoral research associateship with NOAA-Fisheries. Each row represents a dataset used to update an ecosystem model. I calculated the number of days it took me to get various datasets for the ecosystem model I was tasked to update by subtracting the date I initially sent an e-mail to data managers from the date that I received the final dataset for use in the model. The number of total e-mails sent by all parties, and the hours of meetings spent discussing the data and/or the transfer of data were counted. The last row is a openly available dataset [101].

initial email	final data	# days	# emails	hours of meetings
12 05 2021	25 04 2022	348	70	2
05 04 2021	27 01 2022	297	152	11
21 04 2021	12 07 2021	82	73	2
24 06 2021	02 08 2021	39	99	3
12 05 2021	04 06 2021	23	60	3
10 05 2021	10 05 2021	0	103	3
01 05 2021	03 05 2021	2	101	3
—	—	0	0	0

I am not alone in realizing the inefficiencies and difficulties in obtaining data from authors [106–109]. Bledsoe *et al.* [110] illustrate other examples of difficulty in rescuing data; for example, they highlight that after 5 years of trying to locate data from the monitoring that followed the Exxon Valdez oil spill, researchers were only able to recover 30% of the data, estimating that $105 million USD was spent on collecting data that are no longer available [110]. In general, requests for previously published research are highly unsuccessful, failing 41–73% of the time [106,111–114]. In a particularly stark example, Wicherts *et al*. [112] sent more than 400 e-mails over the course of 6 months and only received data for 27% of the publications of interest (73% of authors did not share). Many researchers are on short-term contracts and are unable to fulfil project goals with such delays, leading to wasted resources and stifled careers. Many organizations around the world do not have adequate funds to support researchers waiting for access to valuable data. Likewise, if we expect to respond to climate change in a reasonable amount of time, a more efficient system would be useful.

Yet, we cannot blame individual researchers for experiencing real barriers to sharing their data and code [29]. Instead, a culture shift is necessary to adopt open science practices [115]. Institutions can support data and code archiving with resources (e.g. funding, formal guidance [116], workshops and training, library staff assistance). For example, the American Geophysical Union has developed and compiled resources as guidance for authors, which are also applicable elsewhere (https://data.agu.org/resources/). While open data mandates are useful in increasing data availability [113], the shared data are often not reusable [117]. Thus, while it is a useful first step for top–down entities to ‘require’ data sharing, compliance may improve with enforcement and quality control. Some authors have suggested that enforcement could include withholding research funding [58,102], while others include delaying promotion or tenure, or holding back journal article publications as potential options [118]. Several funding agencies have explicit open data-sharing policies and mention monitoring compliance and sanctions in cases of non-compliance (e.g. [119], §3). Journals such as *The American Naturalist*, the *Journal of Evolutionary Biology*, *Ecology Letters* and the *Proceedings of the Royal Society B* each have designated data editors whose role is to ensure that authors provide organized data and metadata in long-term repositories for future re-use [120].

The sharing of data and code can and should be streamlined to save costs and reduce waste (e.g. time, resources, funding, etc. [104]). As an example from my own work, the National Oceanic and Atmospheric Administration’s (NOAA-Fisheries) Northwest Fisheries Science Center West Coast Groundfish Bottom Trawl Survey team (last row in table 1) has stored their data in an open and freely available data warehouse that can be pulled directly into R via a custom package [101]. These efforts, while a significant upfront time commitment, will likely continue to provide researchers with instant access to their data, saving everyone (including the original data providers) time and funds in the long term. Imagine the science that could be conducted, the models that could be built and the questions that could be answered if the data that has been collectively gathered were well-documented, organized and immediately accessible (i.e. following FAIR principles) to everyone.

## 3. The collective advantages tomorrow

Open science has already greatly benefitted humans [48,50,121], and there is no reason to believe that further progress on this front will not continue doing so long into the future [49,51,122]. Open data and code will likely allow future scientists the ability to predict, with even greater precision and accuracy, future climate states, and the resulting patterns of animal movement and habitat availability for the world’s species [123,124]. As organism distributions shift on global and regional scales [125–127], species will cross geopolitical boundaries [128,129]. Future scientists will likely have many versions of entire Earth biosphere models, to better predict the management outcomes of harvested species and responses of endangered species under continued changes to our planet. If scientists consistently perform the actions required for achieving open science, it may allow future humans to have access to data that we can only wish that we had today [130,131]. Today’s actions can pave the path for how many centuries-long time-series datasets future humans will inherit. Although it is worth noting that this process will require data curation, including preservation, to ensure these data are usable for the long term [132,133].

Transparent sharing of data with the world can lead to a more inclusive and collaborative future of global natural resource management [134,135]. Many countries and institutions around the world do not have adequate funds to collect data or pay for the use of (or development of) analytical software. While computing power costs can still be prohibitive in some cases, personal computing hardware has become more accessible and cheaper than ever. Combined with freely available data and code, many more students and researchers are empowered to participate when science is open [122,136,137]. This can lead to collective benefits as a diversity of perspectives leads to a diversity of solutions to global issues [138,139].

We are in an era of scientific progress that is highly wasteful [104,140–144]. Individuals tend to cherish the research funds they are granted, but take for granted the monetary value of data and code after they have been collected or created. Collectively, the United States and the European Union spend hundreds of billions of dollars on research [145,146]. Ensuring that these funds are well-spent gathering information or building tools that will have long shelf lives should be of utmost importance [141]. Yet, we continually fail to archive our data and code in long-term repositories [27,77,108,147]. Sutherland [105] suggests that policy and management decisions are often made without regard to the available evidence, such that actions often do not lead to the desired results, and Buxton *et al.* [104] reports that this can lead to the waste of time, funds, and valuable natural resources and ecological services. This begs the question of why scientists collect data in the first place. Creating a culture of open data and code access may lead to more appropriate policies and management practices as decision-making moves towards a more transparent evidence-based process [102,103,134,135]. Instead of pretending that our data are only useful for the research question at hand, adopting the mindset of assuming that our data are valuable to the world might lead to better open science practices.

In 2008, the Svalbard Global Seed Vault was opened. Its purpose is to ‘… secure the foundation of our future food supply’ [148]. As of September 2023, 99 organizations have contributed over 1.2 million seed samples since the Global Seed Vault opened [148]. This highly collaborative effort prioritizes the future of humanity in an uncertain future. Like the hallways of seed storage shelves in the Seed Vault, hallways of servers are ready to be filled with information for future generations. And similarly, data and code will help ‘… future generations to overcome the challenges of climate change and population growth’ [148]. The question that we must ask ourselves is ‘what insurance policy do we want to have with our global knowledge?’

Large databases that store information compiled from many sources parallel global efforts such as the Seed Vault. One example of these databases is the Ocean Biodiversity Information System (OBIS), which supports over 45 million observations of diverse taxa from bacteria to marine mammals, collected by 500 institutions in 56 countries ([96]; https://obis.org/about/). Considerable collaborative effort has gone into the standardization and integration of such a database, including delivery to the Global Biodiversity Information Facility (http://www.gbif.org), for the benefit of those wishing to access such data [149]. Curating such large databases is no simple feat and requires additional effort beyond the initial data sharing by the original researchers. For example, changes to taxonomic nomenclature over time, typos and other errors pose serious challenges to the organization of these databases.

OBIS devotes significant staff time to helping clarify the appropriate metadata, which importantly takes some burden off of the researchers who collected the primary data, as they are guided through this process [149]. Yet, in general, databases often lack appropriate metadata, which hinders the find-ability and re-usability of available data [150–152]. Differences in methodology, ontology and definitions of vocabulary can lead to a lack of interoperability between datasets. While there have been many efforts to standardize metadata (e.g. [67–69,152]), these standards are lacking in many disciplines and are not adequately policed in many repositories. Institutions, funding agencies and grant writers can play a role by providing monetary and logistical support for such efforts. The costs of maintaining these databases are substantial but are outweighed by the benefits to scientific progress and society [153–158].

## 4. A change in culture

What is the unit of knowledge that we would most like to protect for future generations? Is it the scientific publication? Or is it our datasets? Datasets are snapshots in space and time of *n*-dimensional hypervolumes of information that are resources in and of themselves—each giving numerous insights into the measured world [134,135]. New publishing paradigms, such as Octopus, allow researchers to link multiple ‘Analysis’ and/or ‘Interpretation’ publications to a single ‘Results’ publication as alternative analyses and interpretations of the same data [159]. A more traditional research paper, on the other hand, is one realization of many possible assessments of the data that were originally collected, and a wide diversity of results can be obtained when many individuals analyse one dataset with the same research question in mind [160,161]. That is, publications are one version of an oversimplified projection through *n*-dimensional space which communicate stories that our human minds can comprehend. Manuscript narratives, by necessity, leave out information to craft such a story.

This is not to say that scientific publications in and of themselves are not useful. On the contrary, they frame our current and historical understanding of the world and put scientific inquiry into the relevant spatial and temporal context. Scientific articles offer analysis and interpretation of data which will allow future generations to understand why certain policies, management actions, or approaches were attempted and/or abandoned. However, if future researchers are not granted access to our (past) data, future humans will have to repeat costly (e.g. time and resources) experiments, laboriously extract information directly from figures, tables and text in the articles themselves (assuming the relevant information is available and detailed enough, although there is evidence that this is not the case in at least some disciplines [55,162]) or will have to trust our analytical procedures and our intuitions and perceptions about the data we collected [160,161].

We do not hesitate to spend our valuable time and resources to publish our manuscripts in journals, have DOIs minted and collectively fund the hosting of these research products in multiple archives. Yet, we argue that a lack of time and funding keeps us from doing the same with our datasets [27]. We need to consider datasets a basic unit of knowledge, the importance of which is arguably at least on par with the importance of storing the articles that are written about them [134]. Fortunately, we do not have to choose between the two. We can archive both if we choose to.

The mind-bendingly fast growth of artificial intelligence is changing the scale and scope of what we can learn with the information around us. Who can possibly know what statistical methodology or machine learning algorithms we will be applying to our data in 2100? It seems nearly certain that scientists in 2100 will rather have access to our data than any antiquated model output or table of summary statistics that we might generate today. Our ‘data available upon request’ statements, which are still pervasive in our manuscripts [106–109], will give future scientists a bit of frustration with our lack of foresight, as this type of data availability declines rapidly with age [163]. What computer, hard drive, institutional e-mail address, or contemporary researcher will be alive and operational in 2100 to share their data upon request?

As knowledge workers, we should strive to show our work—archive our data and code—to safeguard the uncertain future of our species. Earth contains complex, highly dynamic systems that we often make sense of with access to high-quality data [164] and complex modelling [95,165,166]. Society collectively benefits from openly sharing data and software with one another today and tomorrow as we form a better understanding of the world around us—a world that humans rely on for survival. Open science practices broaden participation in science and create a more inclusive, equitable and sustainable world as useful information (e.g. data and code), and a diversity of solutions, are shared—including with those who cannot afford to collect costly data [75,167]. As the cost of computational resources continues to decline, the usefulness of open data and code to underfunded scientists around the world only continues to increase. By locking information behind paywalls and taking (taxpayer-funded) datasets to the grave, knowledge workers exclude most of the world’s population from the process of science and the state of the resulting knowledge. Alternatively, investing in open science today can help to ensure information is available for unborn generations.

## Data Availability

This article has no additional data.
